# Two sides of a coin: Effects of climate change on the native and non-native distribution of *Colossoma macropomum* in South America

**DOI:** 10.1371/journal.pone.0179684

**Published:** 2017-06-27

**Authors:** Taise M. Lopes, Dayani Bailly, Bia A. Almeida, Natália C. L. Santos, Barbara C. G. Gimenez, Guilherme O. Landgraf, Paulo C. L. Sales, Matheus S. Lima-Ribeiro, Fernanda A. S. Cassemiro, Thiago F. Rangel, José A. F. Diniz-Filho, Angelo A. Agostinho, Luiz C. Gomes

**Affiliations:** 1 Programa de Pós-Graduação em Ecologia de Ambientes Aquáticos Continentais, Universidade Estadual de Maringá, Maringá, PR, Brazil; 2 Programa de Pós-Graduação em Sistemas Costeiros e Oceânicos, Universidade Federal do Paraná/CEM, Pontal do Paraná, PR, Brazil; 3 Universidade Federal do Piauí, Departamento de Biologia, Picos, PI, Brazil; 4 Laboratório de Macroecologia, Universidade Federal de Goiás, Jataí, GO, Brazil; 5 Departamento de Ecologia, ICB, Universidade Federal de Goiás, Goiânia, GO, Brazil; 6 Núcleo de Pesquisas em Limnologia, Ictiologia e Aquicultura (NUPÉLIA), Universidade Estadual de Maringá, Maringá, PR, Brazil; University of Sydney, AUSTRALIA

## Abstract

Climate change and species invasions interact in nature, disrupting biological communities. Based on this knowledge, we simultaneously assessed the effects of climate change on the native distribution of the Amazonian fish *Colossoma macropomum* as well as on its invasiveness across river basins of South America, using ecological niche modeling. We used six niche models within the ensemble forecast context to predict the geographical distribution of *C*. *macropomum* for the present time, 2050 and 2080. Given that this species has been continuously introduced into non-native South American basins by fish farming activities, we added the locations of *C*. *macropomum* farms into the modeling process to obtain a more realistic scenario of its invasive potential. Based on modelling outputs we mapped climate refuge areas at different times. Our results showed that a plenty of climatically suitable areas for the occurrence of *C*. *macropomum* occurrence are located outside the original basins at the present time and that its invasive potential is greatly amplified by fish farms. Simulations of future geographic ranges revealed drastic range contraction in the native region, implying concerns not only with respect to the species conservation but also from a socio-economic perspective since the species is a cornerstone of artisanal and commercial fisheries in the Amazon. Although the invasive potential is projected to decrease in the face of climate change, climate refugia will concentrate in Paraná River, Southeast Atlantic and East Atlantic basins, putting intense, negative pressures on the native fish fauna these regions. Our findings show that short and long-term management actions are required for: i) the conservation of natural stocks of *C*. *macropomum* in the Amazon, and ii) protecting native fish fauna in the climate refuges of the invaded regions.

## Introduction

Climate change and species invasion are widely recognized as grievous threats to biodiversity, generating great conservation and socio-economic demands worldwide [1, 2, 3, 4, 5]. It is crucial to understand the interactions between climate change and biological invasions, since the impacts caused are progressively increasing worldwide and generating negative changes in native communities [6, 7]. For this reason, there is a growing consensus that management decisions aimed at biodiversity conservation should be made.

Freshwater environments play an important role in determining global biodiversity and providing valuable goods and services for humans [8, 9]. Despite their importance, these ecosystems are subject to unprecedented levels of anthropogenic impacts, among which invasive species occupy a central position in the level of threat [10]. Freshwater ecosystems are also particularly vulnerable to climate change, because increases in temperature and change in precipitation regimes affect the water runoff dynamic, seasonality and duration of floods and droughts, water temperature and water quality [11, 12, 13]. Thus, there is an emergent need for government policies and management actions to protect freshwater ecosystems and their species, taking into account the coupled effect of invasions and climate change.

Fish species have been continuously introduced on a global scale, with invasive species now occupying a large number of drainage basins at an unprecedented rate [14, 15, 16]. Fish invasions promote impacts at population, community and ecosystem levels primarily due to competition, predation and changes in the structure and quality of habitats, hence magnifying the impacts of other anthropogenic assaults [17, 18]. Among the causes of freshwater fish introductions, fish farming stands as the main driver in the Neotropical region [17, 19]. In aquaculture, the escape of non-native species represents a constant source of propagules, increasing colonization pressure, thus facilitating species invasion [20, 21, 22, 23].

In South American basins, introduced fish species, both from South America itself (allochthonous invaders) and from other continents, are commonly used in fish farming. Many of these fish species have become invasive, and some are starting to colonize non-native areas [24]. Because there is no fully safe confinement in aquaculture, the flow of non-native propagules into different drainage basins is expected to be constant and intensive [17]. In Brazil, this situation has worsened considerably with government subsidies to rear non-native species in aquaculture cages in reservoirs, from which escapes are inevitable, generating pressures on wildlife [23, 24]. The widespread release and dispersion of non-native fish through climatically suitable areas into South American rivers are major concerns from a conservation viewpoint. Furthermore, global warming may expand the geographic areas with suitable temperatures for aquaculture, boosting the impacts of invasive species [4].

Additionally, climate change has complex effects on the potential distribution of freshwater fish, mainly by altering the environmental conditions where species find the bulk of requirements needed to complete their life cycle in the present-day. The multiple responses to climate change have involved the shift, expansion and contraction of geographical ranges. However, most of the evidences is geographically and taxonomically biased towards the temperate regions and salmonids, respectively [25, 26]. Therefore, the scientific debate is restricted to a limited level in the face of worldwide extraordinarily high freshwater fish diversity worldwide. The gap in knowledge is still more critical when considering the responses of introduced species to climate change, with a scarcity of studies in tropical regions.

*Colossoma macropomum* Cuvier 1818, popularly known as tambaqui, is a native fish species from the Amazon and Orinoco River basins in northern South America [27]. Due to its large body size and highly appreciated meat for human consumption, tambaqui is among the most cultivated Neotropical fish species in fish farms [28]. Even though the escape of *C*. *macropomum* is not systematically monitored, the frequency of such events is high in the face of its continuous and intensive farm production. To date, there is no record of *C*. *macropomum* having formed large non-native populations, and their potential impacts are still not precisely known [17]. However, constant escapes from fish farms and colonization facilitated by climatic suitability may considerably enlarge any *C*. *macropomum* populations in non-native regions, increasing the probability of establishment in different South American river basins, potentially affecting the native fish fauna of such areas.

In this study, we consider *C*. *macropomum* as a potential invasive species for all South American river basins since its barrier to dispersal has been broken by human activities, especially through fish farming in net cages [17, 23]. Relying on correlations between climate and species occurrences our study simultaneously assesses the effects of climate change on the native distribution of *C*. *macropomum* and on its invasiveness across river basins of South America, using an ecological niche modeling approach. First, we estimated the range expansion of the species through climatically suitable areas of the continent at the present time. Then, we assessed the effects of climate change projected for 2050 and 2080 on the geographical distribution of *C*. *macropomum* in native and invaded regions. Finally, we identified climate refugia in the native region, corresponding to strategic areas for implementing species conservation measures, as well as in the invaded regions, corresponding to areas in which native fish fauna should experience the negative effects of *C*. *macropomum* invasion in the face of climate change. In addition to conservation issues, we also addressed considerations on socio-economic issues due to the effect of climate change on distribution of *C*. *macropomum*. The model predictions indicated that favorable climatic conditions, abundant at the present time, would become scarce in the future, implying conservation and socio-economic concerns in the native region. Although the invasive potential of this species is predicted to decrease in the face of climate change, climate refugia will remain in the Paraná River, the East Atlantic basins and the Southeast Atlantic basins, putting intense, negative pressures on native fish fauna.

## Methods

### Occurrence data of the species

The occurrence data of *Colossoma macropomum* in South America were obtained from three databases: FishNet2 (http://www.fishnet2.net/), SpeciesLink (http://splink.cria.org.br/) and Global Biodiversity Information Facility (GBIF; http://www.gbif.org/). We also used occurrence data provided by field samplings carried out by Universidade Estadual de Maringá/ Núcleo de Pesquisas em Limnologia, Ictiologia e Aquicultura–Nupélia, Universidade Federal do Tocantins/ Núcleo de Estudos Ambientais–Neamb and Limnobios. This search was still supplemented with an extensive review in ISI (http://www.isiknowledge.com/) and Google Scholar (http://www.googlescholar.com/) using “*Colossoma macropomum”* as a key word. Occurrences of *C*. *macropomum* in non-native regions of the South America mostly originated from stocking and fish farming. Regarding the latter, fish farms are continuously putting juveniles and adults of *C*. *macropomum* into the natural environments of non-native areas mainly due to: i) the escape of individuals along with the effluent water; ii) their confinement breaking by natural flooding and iii) the inappropriate management of tanks. Thus, fish farming constitutes the main vector of *C*. *macropomum* introduction in South America. Based on the assumptions that there is no fully safe confinement and that fish farms act as constant and effective propagule sources for the cultivated species [17, 19], we added the locations of fish farms rearing *C*. *macropomum* as surrogates of species occurrence in natural environments, along with natural occurrences, to obtain a more realistic scenario of invasive potential of the species in the continent over time.

**Fig 1:**
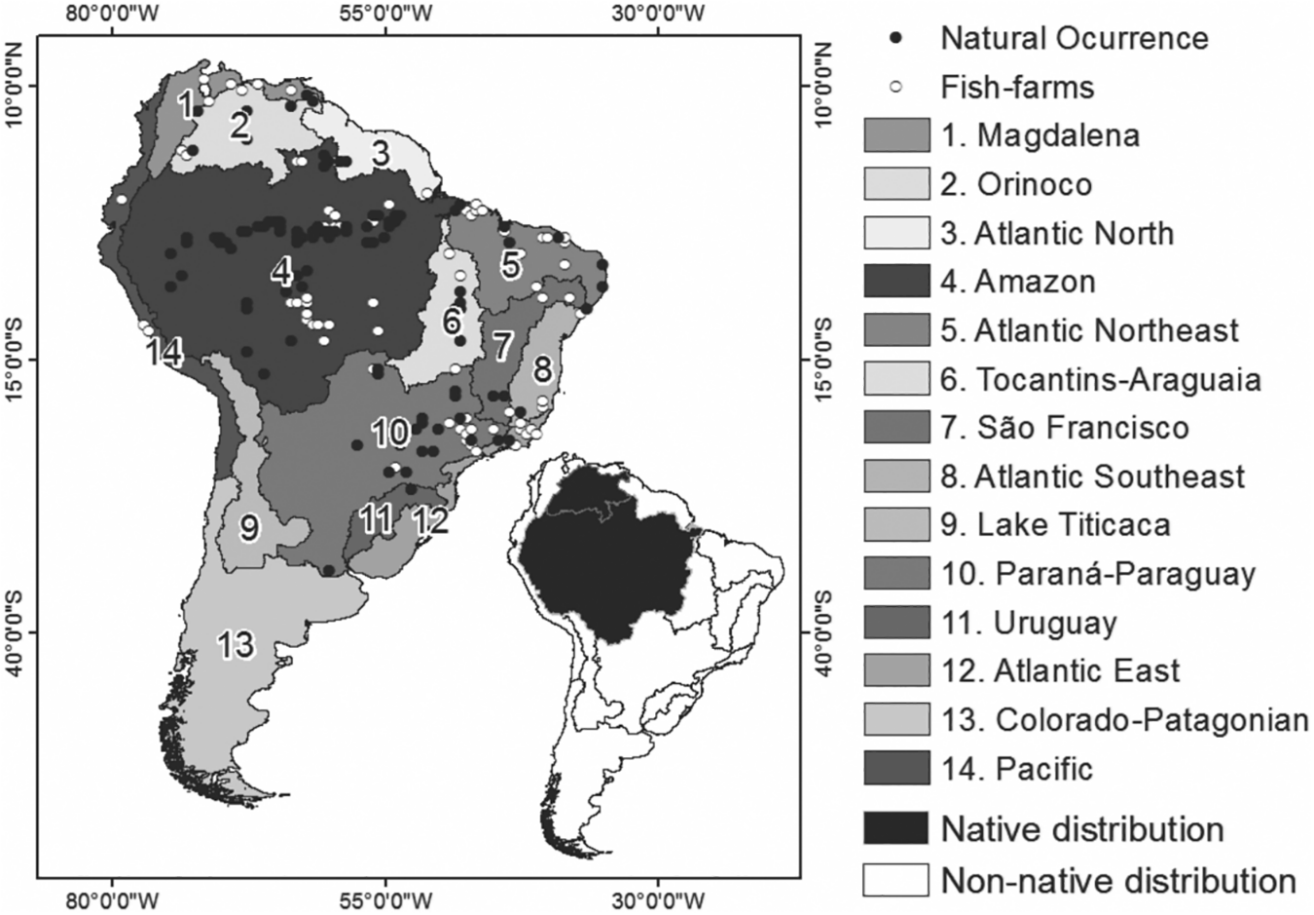
Occurrences of *Colossoma macropomum* in South America. The small map shows the native range.

The occurrence records from both the natural environment and fish farms were mapped on a regular geographical grid, containing 6,180 cells with spatial resolutions of latitude and longitude of 0.5°, representing the 14 major river basins of South America. The area of each cell grid corresponds to approximately 3025 km^2^ (55 x 55 km). The mapping of the occurrence records on the geographical grid resulted in 178 occupied cells. Thus, two binary matrices of presence and pseudo-absence were constructed: (1) South America considering occurrences from natural environments (104 occupied cells) and (2) South America considering occurrences from natural environment and fish farming together (178 occupied cells) (see Fig 1 and S1 Table for details).

### Bioclimatic variables

We used four bioclimatic variables related to the environmental tolerance of the species as predictors of the species distribution: maximum temperature of the warmest month (^o^C; TMAX), minimum temperature of coldest month (^o^C; TMIN), precipitation of the wettest month (mm; PMAX) and precipitation of the driest month (mm; PMIN) [29]. Temperature and precipitation are the major climatic parameters determining the distribution of organisms on Earth. The former is responsible for altering the metabolism (especially the enzymatic activity) of living organisms, and the latter determines the seasonal variations of droughts and floods, synchronizing biological events of the species, such as migration, spawning, home range and growth. We also used two hydrological variables as measures of water availability in each grid cell: the number of rivers (NR) and the upstream flow length (m; UFL, corresponding to distance from the headwater sources) (http://eros.usgs.gov/). In addition to bioclimatic and hydrological variables, we also used mean altitude (m; ALT) as a predictor variable to address distribution restrictions imposed by the Andes, Neblina Peak and Venezuelan Mountains in Northern Amazon and Serra do Mar Mountains in eastern Brazil [29].

Forecasts of future bioclimatic variables (projected for 2050 and 2080) were extracted from the model by the Intergovernmental Panel on Climate Change, Fifth Assessment Report (IPCC-AR5) (http://ccafs-climate.org). We used four Atmosphere-Ocean General Circulation Models (AOGCMs): CCSM (Community Climate System Model), CSIRO (Australia’s Common wealth Scientific and Industrial Research’s General Organization), MIROC (Model for Interdisciplinary Research on Climate) and MRI (Meteorological Research Institute). The concentration trajectory for each AOGCMs was based on the Representative Concentration Pathways 4.5 (RPC 4.5; moderate scenario of carbon emission within the optimistic context). We assumed the ALT, NR and UFL were temporally stationary to perform future predictions.

### Ecological niche modeling

Considering that broad scale patterns of species occurrence are determined by the responses of organisms to different environmental conditions (reflecting the Grinellian component of the ecological niche, *sensu* [30]), we used ecological niche models (ENMs) to predict the effects of climate change on the distribution of *C*. *macropomum*. The two species occurrence matrices (South America considering occurrences in natural environments, and South America considering occurrences in natural and fish farming environments) were modeled with ENMs, using the layers of climatic-environmental variables. The fit of ENMs yield suitability matrices, from which the potential distribution of *C*. *macropomum* was mapped given the present and future climates.

We used six conceptually and statistically different ENM based on presence-only and presence-background data: Bioclim (BIOC; [31], Euclidean distance (EUCD; [32]), Gower distance (GOWD; [32, 33]), Mahalanobis distance (MAHD; [34]), Ecological Niche Factor Analysis (ENFA; [35]), and Maximum Entropy (MAXE; [36]). Predictions vary among ENMs because of the different assumptions of each model. Because all models are biologically plausible, we treated the variation among predictions as statistical uncertainty [37]. To account for the statistical uncertainty and minimize inferential errors, we employed the *ensemble forecasting* approach, which consists of calculating the “consensus” of multiple models (CONS, [38]). Assuming that distinct sources of errors affect each ENM in different ways, by obtaining a consensus model, the errors in each individual prediction tend to cancel each other out, therefore producing a more reliable prediction [39].

For each ENM, the extent of species occurrence was randomly divided into two subsets: 75% for calibration and 25% for evaluation. This procedure was repeated 50 times to avoid bias in the calibration and evaluation of the data sets. We converted the continuous predictions of suitability of each ENM into binary vectors of presences and absences in each cell (1/0) using the threshold that maximizes sensitivity and specificity values in the receiver operating characteristic (ROC) curve. The ROC curve is generated by plotting the fraction of true positives versus the fraction of false positives at various threshold settings. The distribution of *C*. *macropomum* in current climatic conditions was estimated using 300 predictions (6 ENMs x 50 randomizations) for each species occurrence matrix. The simulations for future climatic conditions were estimated obtaining 1200 predictions (6 ENMs x 50 randomizations x 4 GCMs) for each future time (2050 and 2080) and for each specie occurrence matrix. This procedure allowed us to generate a frequency of projections in the ensemble. We then generated the frequency of projections weighted by the TSS statistics for each region and time, i.e., better models have more weight in our consensus projections. The TSS range from -1 to +1, where values equal to +1 are a perfect prediction and values equal to or less than zero are a prediction no better than random [40]. We considered the species present only in cells in which at least 50% of the models retained in the ensemble predicted the species as present.

Principal components analysis (PCA) [41] was used to compare the suitability outputs derived from alternative ENMs and their consensus at current and future times. This analysis allowed us to: i) evaluate the degree to which different ENMs converge in estimates of the climatic suitability of *C*. *macropomum*; and ii) determine which model reflects the main direction of variation among the suitability maps [42]. In our study, only the results of models reflecting the main direction of variation among the suitability maps were interpreted. The suitability of native region was obtained from results generated for 2.429 cells of South America, representing the Amazon and Orinoco basins. Given that in native basins the escapes of *C*. *macropomum* from fish farms do not generate impact, the analyses covering the native region were focused only on natural occurrences, disregarding fish farms.

From the presence and absence outputs provided by ENMs reflecting the main direction of variation of the suitability data, we estimated the native range of *C*. *macropomum* and its expansion across climatically suitable areas of South America. We generated an occupancy matrix for the 14 major South American basins: the Amazon and Orinoco basins representing the native region, and the Pacific, Magdalena, North Atlantic, Northeast Atlantic, Eastern Atlantic, Tocantins-Araguaia, São Francisco, Paraná-Paraguay, Uruguay, Southeast Atlantic, Lake Titicaca and Colorado-Patagonian basins representing the non-native regions. Then, we computed the total number of occupied cells in native and non-native basins at the present time. We analyzed the effects of climate change on native distribution and the non-native regions by assessing the range shift, expansion and contraction phenomena, and performed the same protocol for the two future times. We also performed such analysis considering the natural environmental and fish farming occurrences together, to portray a more pessimistic scenario with respect to the invasion of *C*. *macropomum* into South America river basins. Then, we identified the basins in which the invasive process should be amplified by fish-farm activity over time. In addition, we finally identified the areas of South America serving as climate refugia for *C*. *macropomum* in 2050 and 2080. Climate refugia in the native region correspond to the areas of greatest interest for the conservation of the species and in the non-native regions correspond to the areas in which the native fish fauna has the most potential to experience the impacts of *C*. *macropomum* invasion in the face of climate change.

Ecological niche modeling was carried out in the computational platform BioEnsembles [37] and PCAs were performed in SAM v.4.0 [43]. The maps were produced in ArcGIS v.10.2.

## Results

Our survey indicates that *C*. *macropomum* is common in the invaded regions, especially in the Paraná-Paraguay River basin. Fish farms stocking the species are common in the Paraná-Paraguay, East Atlantic, Northeast Atlantic basins and in the Amazon (Fig 1). Among the 14 South American river basins, the Pacific, Lake Titicaca and Colorado-Patagonian basins do not show occurrences of *C*. *macropomum* in natural environments.

**Fig 2:**
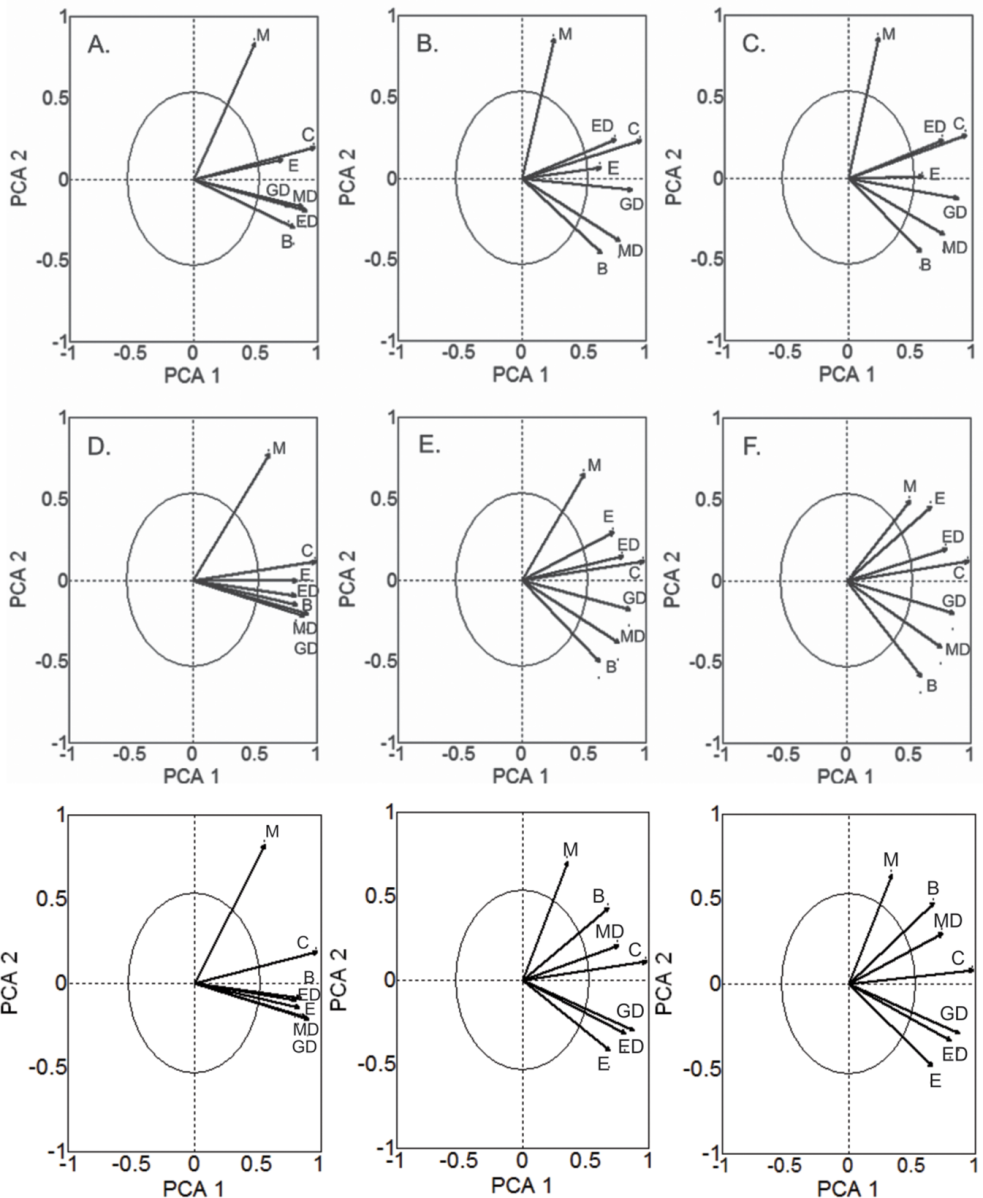
Principal component loadings on the first two axes of PCA representing the suitability of *Colossoma macropomum* for native region at the present time (A), 2050 (B) and 2080 (C); for South America considering natural environment occurrences at the present time (D), 2050 (E) and 2080 (F); and for South America considering the total occurrences (natural environments and fish farming together) at the present time (G), 2050 (H) and 2080 (I). B = Bioclim, ED = Euclidian Distance, GD = Gower Distance; MD = Mahalanobis Distance, M = Maxent; E = Enfa and C = consensus model.

The first two PCA axes represented a large proportion of variation between suitability maps generated by different ENMs. The accumulated proportion of variation represented by the two axes ranged from 69% to 84% in native region in 2080 and South America at the present time, respectively (Table 1). For the present time, the BIOC and distance methods, and ENFA and CONS produced similar suitability predictions (Fig 2A and 2D), with the exception of CONS, wich differed from the others models for South America considering total occurrences (Fig 2G). In the future, the predictions of CONS were more similar to those of EUCD (Fig 2B, 2C, 2E and 2F), except for when CONS was more similar to MAHD for South America considering total occurrences (Fig 2H and 2I). In general, MAXE produced results markedly different from the other methods. The CONS model had the highest loadings for the first PCA axis for different times and data sets (Table 1), reflecting the main direction of variation among suitability maps. Consequently, only the outputs derived from the CONS method were retained for interpretation.

**Table 1:** Values of the PCA loadings of different modeling methods for native region and South America. The “Total” column corresponds to the results of modeling considering natural environmental occurrences along with fish farm occurrences.

	Native region		South America			
	Natural environment		Natural environment		Total	
	PCA1	PCA2	PCA1	PCA2	PCA1	PCA2
**Current time**						
BIOCL	0.81	-0.30	0.84	-0.15	0.85	-0.09
EUCDIST	0.77	-0.16	0.83	-0.097	0.84	-0.14
GOWDIST	0.91	-0.20	0.93	-0.21	0.92	-0.21
MAHADIST	0.88	-0.17	0.89	-0.22	0.89	-0.20
ENFA	0.72	0.12	0.84	<0.001	0.81	-0.09
MAXENT	0.50	0.84	0.62	0.79	0.56	0.82
CONS	**0.98**	0.20	**0.99**	0.11	**0.98**	0.17
Axes explanation (%)	0.65	0.13	0.73	0.11	0.72	0.12
Accum. explanation (%)	0.78		0.84		0.84	
**Future 50**						
BIOCL	0.65	-0.46	0.63	-0.50	0.69	0.43
EUCLDIST	0.76	0.24	0.82	0.14	0.81	-0.32
GOWDIST	0.90	-0.07	0.87	-0.18	0.88	-0.30
MAHADIST	0.79	-0.39	0.78	-0.39	0.76	0.21
ENFA	0.64	0.07	0.74	0.30	0.69	-0.42
MAXENT	0.26	0.86	0.51	0.65	0.35	0.71
CONS	**0.96**	0.23	**0.99**	0.11	**0.98**	0.11
Axes explanation (%)	0.54	0.17	0.60	0.14	0.58	0.16
Accum. explanation (%)	0.71		0.74		0.74	
**Future 80**						
BIOCL	0.59	-0.46	0.60	-0.59	0.68	0.48
EUCLDIST	0.76	0.24	0.81	0.20	0.81	-0.33
GOWDIST	0.88	-0.13	0.86	-0.2	0.88	-0.29
MAHADIST	0.78	-0.35	0.76	-0.41	0.74	0.3
ENFA	0.60	<0.01	0.68	0.46	0.65	-0.48
MAXENT	0.25	0.87	0.51	0.49	0.34	0.65
CONS	**0.96**	0.27	**0.99**	0.12	**0.98**	0.08
Axes explanation (%)	0.52	0.17	0.58	0.15	0.56	0.17
Accum. explanation (%)	0.69		0.73		0.73	

**Fig 3:**
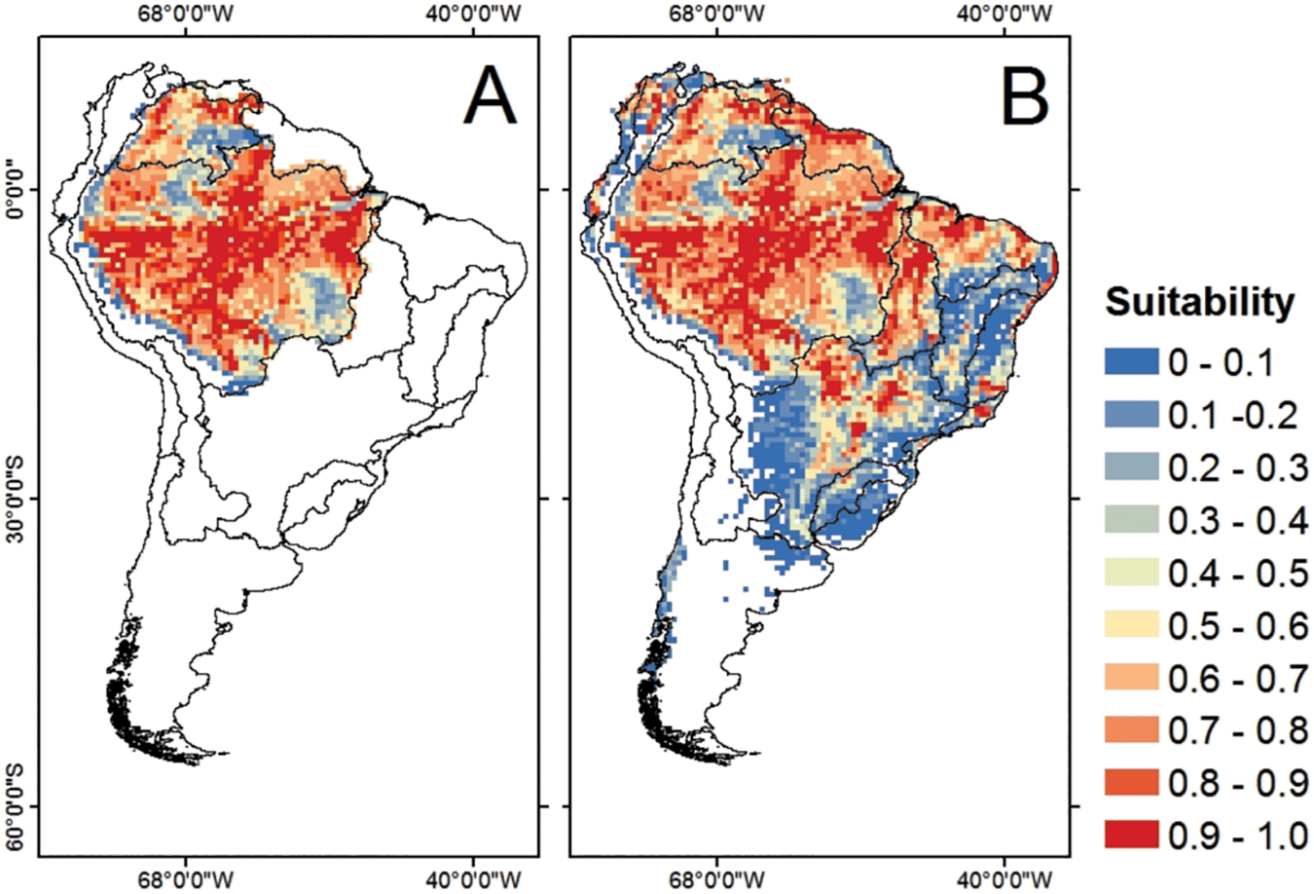
Habitat suitability for *Colossoma macropomum* occurrence derived from the consensus model for the native region (A) and South America (B) at the present time.

The results of the climatic suitability of the CONS model pointed out the northern and northwestern portions of the native region as unsuitable for *C*. *macropomum* (Fig 3A). Amazonian Rivers, especially those located in the southern portion (from southwestern to southeastern), hold the most suitable environmental conditions in the native region. When the model is extended to South America, a large portion of climatically suitable areas (CSAs) were identified outside the original basins, mainly in the North Atlantic, Northeast Atlantic, Tocantins-Araguaia and Paraná-Paraguay (Fig 3B). The species distribution in the native region was estimated to be 1,668 cells, corresponding to approximately 68.67% of the original basins (Table 2). By transposing geographical barriers, it is expected that this species will expand its range by 999 cells (corresponding to approximately 59.9% of their native range), occupying approximately 24.6% of the invaded region. Our results show that the invasive potential of *C*. *macropomum* is greatly amplified when the locations of fish farms rearing the species are included in the modelling process. Because the regular introduction of *C*. *macropomum* in contiguous aquatic bodies is just a matter of time, fish farming activity tends to exceptionally magnify the species invasion in the São Francisco (355.6%), East Atlantic (261.5%) and Southeast Atlantic (263.0%) basins. In general, the invasion potential of *C*. *macropomum* expands to 1,318 cells (approximately 32.5% of invaded region) when we consider the natural environment and fish farming occurrences together in the modeling process (Table 2).

**Table 2:** Geographical distribution (number of occupied cells) of *Colossoma macropomum* in South American basins. The “Total” columns correspond to the modeling results considering natural environmental occurrences along with fish farm occurrences.

	Current time				Future Climate (2050)				Future Climate (2080)			
	Natural environment		Total		Natural environment		Total		Natural environment		Total	
	Range (cells)	% occupied	Range (cells)	% occupied	Range (cells)	% occupied	Range (cells)	% occupied	Range (cells)	% occupied	Range (cells)	% occupied
Amazon	1482	69.97			710	33.52			424	20.02		
Orinoco	213	57.1			59	15.82			34	9.12		
*Native region*	1668	68.67			759	31.25			452	18.61		
East Atlantic	13	5.99	34	15.67	22	10.14	34	15.67	24	11.06	35	16.13
Northeast Atlantic	208	57.94	254	70.75	57	15.88	76	21.17	29	8.08	50	13.93
North Atlantic	194	86.61	203	90.63	120	53.57			78	34.82		
Southeast Atlantic	27	14.36	71	37.77	35	18.62	72	38.38	41	21.8	73	38.83
Colorado-Patagonian												
Magdalena	73	33.95	92	42.79	49	22.79			37	17.21	38	17.67
Pacific	32	10.42	38	12.37	22	7.16	25	8.14	18	5.86	24	7.82
Paraná-Paraguay	248	22.94	344	31.82	194	17.95	216	19.98	153	14.15	157	14.52
São Francisco	27	10.23	96	36.36	7	2.65	27	10.23	3	1.14	21	7.95
Titicaca												
Tocantins-Araguaia	214	66.88	251	79.06	42	13.13			10	3.13		
Uruguay	5	3.42			24	16.44			22	15.07		
*Invaded region*	999	24.65	1318	32.52	551	13.59	576	14.21	397	9.8	414	10.21

**Fig 4:**
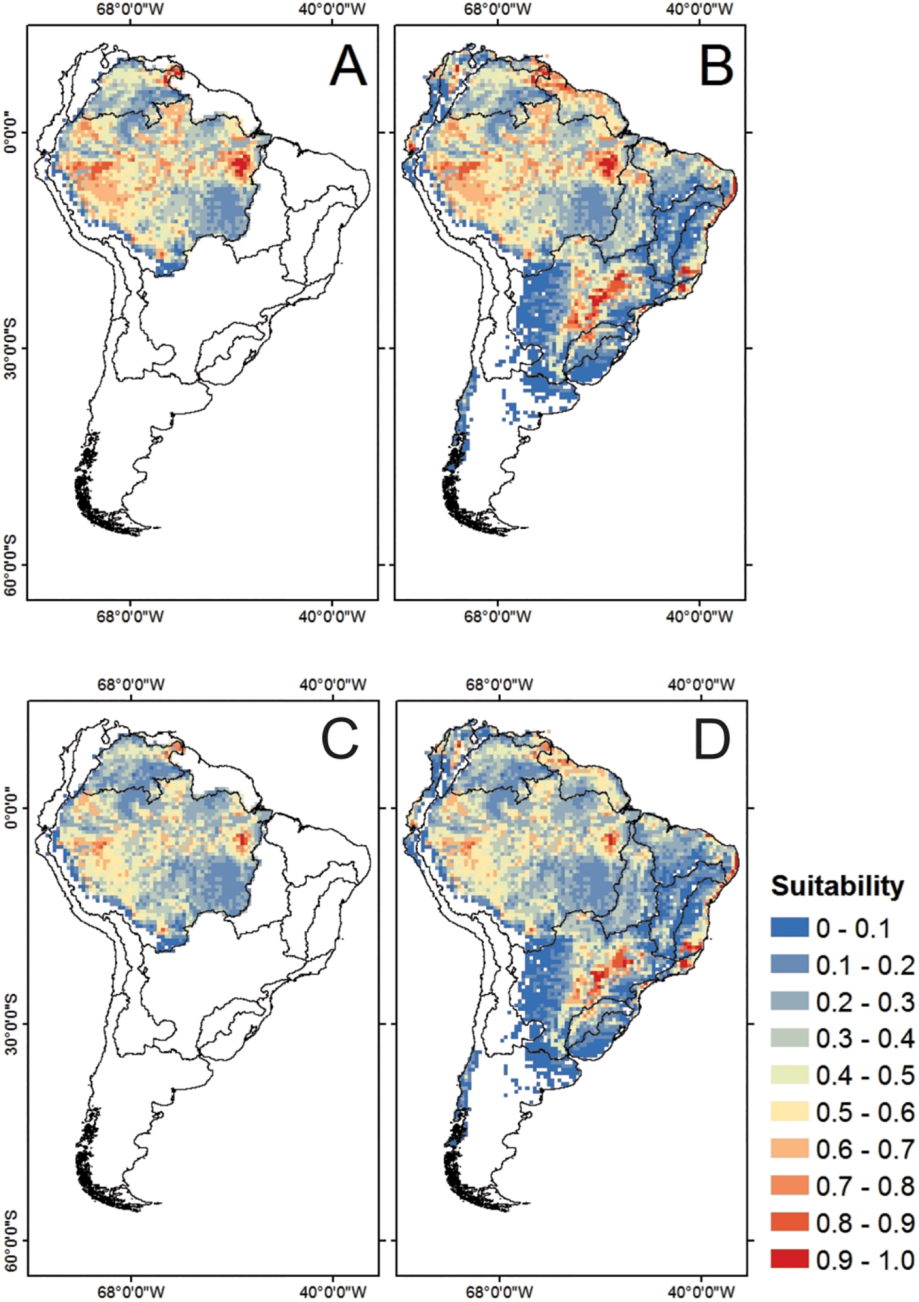
Habitat suitability for *Colossoma macropomum* derived from a consensus model for the native region (A) and South America (B) in 2050, and for the native region (C) and South America (D) in 2080.

In general, our study revealed marked losses of climatically suitable areas for *C*. *macropomum* in the face of climate change. The models showed small portions of highly suitable areas for the occurrence of the species in the eastern Amazon projected for 2050 (Fig 4A) and 2080 (Fig 4C). However, a greater amount of highly suitable areas, in the face of climate change, was concentrated outside the native region, especially in the central portion of the Upper Paraná River basin and in the East and Southeast Atlantic basins (Fig 4B and 4D).

Future simulations reveled a range shift accompanied by range contraction phenomena in both the native and invaded regions. For the native region, of the 1,668 cells occupied at the present time, only 759 and 452 should remain occupied in 2050 and 2080, respectively (Table 2). In the invaded regions, the number of occupied cells at the present time (999) falls to 551 and 397 in 2050 and 2080, respectively, evidencing a marked reduction of the invasive potential of *C*. *macropomum* in South American river basins in the face of climate change. When fish farming data is considered, the losses are a little milder, with 576 cells and 414 cells remaining occupied in 2050 and 2080, respectively.

**Fig 5:**
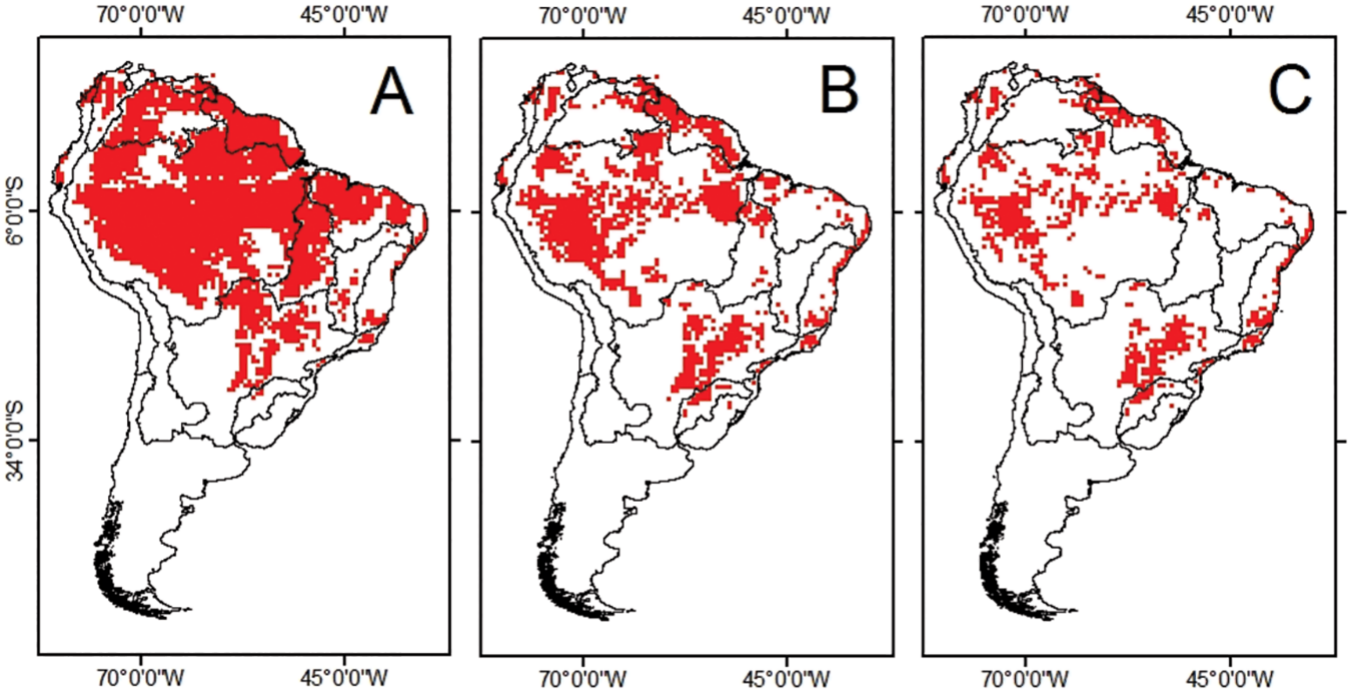
Climate change refugia of *Colossoma macropomum* in South America at present time (A), 2050 (B) and 2080 (C).

Despite the wide geographical range of the species in South America at the present time (Fig 5A), our future projections pointed out that major climate refugia in 2050 should be concentrated in the western, northern and eastern regions of the Amazon, as well as the North Atlantic, East-Southeast Atlantic and Paraná-Paraguay basins (Fig 5B). In 2080, the climate refugia correspond to such regions, albeit with areas that are more restricted with the exception of the East-Southeast Atlantic and Paraná-Paraguay basins (Fig 5C). It is valid to stress that the Paraná-Paraguay basin (the portion of the upper Paraná River, specifically) is the climate refugia that will hold the largest amount of CSAs for the occurrence of *C*. *macropomum* (Fig 4B and 4D).

## Discussion

It is agreed that climate change and species invasions independently cause serious ecological damage. Given that these two continuous phenomena are expected to interact in nature, we assessed the effects of environmental change drivers on the distribution of *C*. *macropomum* in both native and non-native ranges, contributing to bridging the gap of knowledge on the responses of tropical fish species. We show that potential conservation conflicts in the native region are expected as a result of climate change due to extraordinarily large losses of suitable areas. In addition, we call attention to the need for effective strategies against the invasion of this species in climate refugia outside the native region.

Our results indicate low climatic suitability in stretches of the original basins, corresponding to the Venezuelan Amazon and upper Orinoco river regions. Previous studies suggest that a spatially irregular or low sampling effort can bias the distribution modeling results, in particular for sub-regions of the geographic space [44, 45]. However, a study involving extensive samplings of freshwater fish in the Venezuelan Amazon and upper Orinoco basins (269 sampled sites between 1984 and 1999), did not capture any individuals of *C*. *macropomum* (see [46]), inferring support for the climatic unsuitability of this region for this species.

By simulating the potential distribution of *C*. *macropomum* throughout South American basins, we showed that large areas are suitable for the species outside its native basins, especially in the North Atlantic, Northeast Atlantic, Tocantins-Araguaia and Paraná-Paraguay basins. Boosted by stocking and fish farming activities, this species has been successfully in occupying such areas. Since the first half of the twentieth century, stocking activities have been conducted in Northeast Brazil, aiming to increase the region’s fishery production [17]. Such practice was expanded to other non-native basins, and stocking programs were carried out by the Brazilian hydroelectric sector through legal constraints imposed by the organizations of fishery development. The first Fishery Code of Brazil (Decree-Law N° 794 from 19/10/1938), which made fish farming compulsory in reservoirs, is an example of such development policy [17, 23, 47]. In addition, recent government policies have encouraged fish culture in cages placed in reservoirs throughout Brazil, as part of the social programs for food production [17, 23, 48]. Thus, many non-native species have been massively introduced into diverse Brazilian rivers, with *C*. *macropomum* one of the most used Amazon species.

In addition to the massive fish-culture in reservoirs, non-native species have been largely cultivated for either sale or recreational fishing in tanks excavated near the margin of water bodies, especially in densely populated areas [19]. In this sense, by using fish farm locations as proxies of species occurrence, we showed that fish farming tends to magnify the invasion of *C*. *macropomum*, especially in the São Francisco, East Atlantic and Southeast Atlantic basins, which, along with the upper Paraná River, are the most populated basins. Among the biological invasion hypotheses, the human activity hypothesis predicts that antropogenically altered ecosystems (here represented by highly populated river basins) or ecosystems with high propagule pressure (here represented by the water bodies supplying fish farms) make colonization and establishment easier for non-native species [49]. Thus, a positive relationship is expected between non-native species and variables linked to habitat alteration and propagule pressure [49]. Therefore, the human activity hypothesis can be used for the explanation of the invasion process of *C*. *macropomum* addressed here.

By projecting the potential distribution of *C*. *macropomum* for the years 2050 and 2080, under a low-moderate scenario, we observed that the species tends to drastically reduce its geographical distribution in the native region. In general, studies support the idea that the range contraction phenomenon is a response of cold-water fish species to climate change, whereas warm-water species tend to expand their ranges (see [25, 26, 50] for reviews about the effects of climate change on freshwater fish). However, the available information is highly biased towards temperate regions and ‘iconic’ species (e.g. salmonids), thus limiting robust generalizations [25, 51]. This gives relevance to the results presented here, which showed that fish species from warm areas could also experience range contraction facing future climatic alterations.

The habitat unsuitability of the native region for *C*. *macropomum* in the future with consecutive range contraction is not a concern only for the species’ conservation but also from a socio-economic point of view. It is expected that habitat unsuitability will decrease the intrinsic rate of population growth, leading to extinction scenarios over time, thus jeopardizing the species conservation. In addition, the population decline of the species in the native basins tends to cause strong damages to the economy due to its importance in the regional fishery (see [52, 53]). This species has been exploited commercially in the Amazon Basin since at least the end of the nineteenth century, becoming the main species of fishery landings (see [52, 53]), and the preferred species for local consumption [54]. We believe that this scenario can be replicated to other species of economic interest, causing a deep impact in the way of life of the local human population.

When the analyses are expanded to the invaded region, it was shown that climate change tends to considerably reduce the invasive potential of *C*. *macropomum* in South American river basins. The opposite view, i.e., that global warming will exacerbate the threat posed by invasive species, has been widespread in the literature [55, 56]. However, some studies have shown that climate change can reduce invasion risks, especially in lower-latitude regions, which is consistent with our results. In South Africa, 30 invasive grass species should experience the loss of approximately 50% of their potential range by mid-century [57]. In Australia, hawkweed and other weed species are predicted to substantially contract their ranges [58, 59]. With respect to stream fish, invasive species in Australia are predicted to experience both contraction (*Salmo trutta* and *Oncorhynchus mykiss*) and expansion (*Gambusia holbrooki* and *Misgurnus anguillicaudatus*) of their ranges [60].

Although our model has pointed out drastic reduction in distribution of *C*. *macropomum* in both native and invaded regions based on variations in temperature and rainfall dynamics, it is possible that physiological mechanisms, which were not evaluated here, can act to minimize the range losses. Wide tolerance to temperature increases, low concentrations of gases and high concentrations of toxins are traits that should alleviate the climate change effects on aquatic organisms. Only high water temperatures (approximately 35°C) can be considered critical for *C*. *macropomum* [61]. A study found that individuals with a body weight above 250 g have tolerance to low oxygen concentrations due to high temperatures [62]. In addition, the ability of *C*. *macropomum* to obtain O_2_ from an oxygen-rich air-water interface [63] may attenuate the effects of low concentrations of oxygen expected with global warming. However, it is also expected that higher temperatures increase bacterial nitrification in the water, also increasing the metabolic needs of the fish and accumulating NO_2_ in the blood and tissues, which is toxic to fish [64]. *Colossoma macropomum* has a high sensitivity to NO_2_, which makes increases in water temperature potentially harmful for its homeostasis, both in natural environments and fish farming [65]. Thus, a mechanistic approach of ecological niche modeling including adaptive characteristics could be useful to obtain more details about the effect of climate change on *C*. *macropomum* invasion (see [66, 67] for details about mechanistic models).

We showed that climate refugia for *C*. *macropomum* in the invaded regions will be concentrated, especially in the central portion of the upper Paraná River basin and in the East-Southeast Atlantic basins. Habitat suitability may lead to an increase in the population abundance at exceptional levels in the climate refuge regions. Thus, besides having address with restrictions imposed by climate change, the native fauna of such regions should also suffer with the intensification of impacts provided by the invasion of *C*. *macropomum*. The species is an omnivorous fish with a tendency to eat zooplankton, fruits and seeds [68, 69]. Although the competition pressure of these allochthonous invaders might not be severe, omnivorous fish may have as much impact as predator species in native assemblages, as these species are capable of changing environmental quality and nutrient cycling, thus imposing impacts at the ecosystem level [70, 71]. Thus, it is possible that *C*. *macropomum* exerts diffuse effects on native species of fish communities.

Common terminology used for invasive species (see [72]) has led to the feeling that only organisms relocated from great distances (e.g., other continents) can be considered as invaders [73]. This has led to the common belief that species translocated from the same country, such as *C*. *macropomum*, do not cause negative impacts on ecosystems. Thus, a great part of the efforts of scientific research, mitigative measures and public policy does not consider native South American species as invaders in different basins within the continent. This is a misconception, since the introduction of any species outside their natural habitat can cause major disturbances in the native communities [6, 74]. Thus, there is a scientific challenge in making explicit the risks of cultivation of wrongly considered native species explicit.

The drastic changes in the distribution of *C*. *macropomum* in the Amazon and Orinoco River basins are a clear sign that actions for the conservation of the species are urgent. Thus, the maintenance of the original characteristics of climate refugia in the Amazon is crucial. In addition, an important step towards more effective conservation measures would be combining, with our findings, the results for other species in order to identify sub-regions in these watersheds that maximize species occupancy under future climate conditions, and then make them protected areas. It is also noteworthy that the purposes of the National Energy Expansion Plan reveal the intention of the Brazilian government to implement major hydroelectric projects in the Amazon [23], which will especially imperil migratory fish such as *C*. *macropomum*. Thus, it is crucial to develop a detailed delineation of dam placement in the Amazon basin [75], as well as consider the issues raised in this paper. Although we showed a decrease of in the invasive potential of *C*. *macropomum* in South America, the fact that future populations should concentrate in the upper Paraná River, Southern Atlantic and East Atlantic basins, highlights the need to implement short and long-term strategies to control invasion effects for the conservation of native species. The creation of basic specific criteria for the development of fish farming activities using native species, and the acquisition of specific information on the impacts of non-native species, are critical for the success of management measures in the future.

Farming non-native species is a potential cause of biological invasion, and therefore constitutes a significant threat to freshwater biodiversity. Our study suggests that managers and decision makers must carefully define more appropriate long-term strategies for conservation of freshwater biodiversity. These strategies should include preventive management actions and conservation policies that are specifically designed based on the biogeography of species and environmental characteristics of each river basin.

## Supporting information

S1 Table Baseline data of the *Colossoma macropomum* occurrences. (DOCX)
