# Supplementary material for: Two sides of a coin: Effects of climate change on the native and non-native distribution of Colossoma macropomum in South America
Source: PLoS One. 2017 Jun 27;12(6):e0179684. doi: 10.1371/journal.pone.0179684 (PMC5487012; doi:10.1371/journal.pone.0179684)
Supplement: S1 Table — (DOCX) [file pone.0179684.s001.docx]

**Supporting Information**

Two Sides of a Coin: Effects of Climate Change on the Native and Non-native Distributions of *Colossoma macropomum* in South America

Effects of Climate Change on Distribution of *Colossoma macropomum*

Taise M. Lopes^1^*, Dayani Bailly^1^, Bia A. Almeida^1^, Natália C. L. Santos^1^, Barbara C. G. Gimenez^1,2^, Guilherme O. Landgraf^1^, Paulo C. L. Sales^1,3^, Matheus S. Lima-Ribeiro^4^, Fernanda A. S. Cassemiro^1^, Thiago F. Rangel^5^, José A. F. Diniz-Filho^5^, Angelo A. Agostinho^1,6^, Luiz C. Gomes^1,6^

1. Programa de Pós-Graduação em Ecologia de Ambientes Aquáticos Continentais, Universidade Estadual de Maringá, Maringá, PR, Brazil

2. Programa de Pós-Graduação em Sistemas Costeiros e Oceânicos, Universidade Federal do Paraná/CEM, Pontal do Paraná, PR, Brazil.

3. Universidade Federal do Piauí, Departamento de Biologia, Picos, PI, Brazil.

4. Laboratório de Macroecologia, Universidade Federal de Goiás, Jataí, GO, Brazil.

5. Departamento de Ecologia, ICB, Universidade Federal de Goiás, Goiânia, GO, Brazil.

6. Núcleo de Pesquisas em Limnologia, Ictiologia e Aquicultura (NUPÉLIA), Universidade Estadual de Maringá, Maringá, PR, Brazil.

*Corresponding author

[taisemlopes@gmail.com](mailto:taisemlopes@gmail.com)

**Methods**

The occurrence data of *Colossoma macropomum* was supplemented with a review in ISI (http://[www.isiknowledge.com](http://www.isiknowledge.com)/) and Google Scholar (http://[www.googlescholar.com](http://www.googlescholar.com)/) using “Colossoma macropomum” as key word. The review was conducted in August 2015. All articles and thesis obtained from the research were evaluated. We also used occurrence data provided by field samplings carried out by Universidade Estadual de Maringá/ Núcleo de Pesquisas em Limnologia, Ictiologia e Aquicultura – Nupélia, Universidade Federal do Tocantins/ Núcleo de Estudos Ambientais – Neamb and Limnobios. When the occurrences of species were obtained from sampling, they were designated as “natural occurrence”. When the occurrences were obtained from farms, they were designated as “fish farming occurrence”. These occurrences were determined from the geographical coordinates or locations (city and country) informed by the scientific works.

**Results**

The literature review in research bases obtained 107 scientific works, including articles and thesis, and seven occurrences provided by field sampling carried out by universities and environmental consulting. This review obtained 51 “natural occurrences” and 82 “fish farm occurrences” (S1_Table 1).

S1 Table. Baseline data of the *Colossoma macropomum* occurrences.

| **Longitude** | **Latitude** | **Natural Occurrences** | **Fish Farm Occurrences** | **References** |
| --- | --- | --- | --- | --- |
| -79.1 | -0.3 | 0 | 1 | [1] |
| -77.1 | -11.8 | 0 | 1 | [2] |
| -76.6 | -12.3 | 0 | 1 | [3] |
| -74.6 | -8.3 | 1 | 0 | [4, 5] |
| -74.6 | -5.3 | 1 | 0 | [6, 7] |
| -73.6 | 4.2 | 0 | 1 | [8] |
| -73.1 | -3.8 | 1 | 0 | [9, 4, 5] |
| -73.1 | 3.7 | 0 | 1 | [10] |
| -72.6 | 4.2 | 1 | 0 | [11] |
| -71.6 | 10.7 | 0 | 1 | [12] |
| -71.1 | 8.7 | 0 | 1 | [13] |
| -70.1 | -4.3 | 1 | 0 | [4] |
| -69.6 | -4.3 | 1 | 0 | [5] |
| -69.1 | -4.8 | 1 | 0 | [14] |
| -69.1 | 10.2 | 0 | 1 | [15] |
| -68.1 | -3.3 | 1 | 0 | [14] |
| -68.1 | 9.7 | 0 | 1 | [16] |
| -67.6 | -14.3 | 1 | 0 | [17] |
| -66.6 | 10.2 | 0 | 1 | [18] |
| -66.1 | -16.3 | 1 | 0 | [17] |
| -65.6 | -2.3 | 1 | 0 | [19] |
| -64.6 | -3.3 | 1 | 0 | [4, 20] |
| -64.6 | -2.8 | 1 | 0 | [14, 21] |
| -64.1 | -8.8 | 1 | 0 | [4] |
| -63.6 | -13.3 | 1 | 1 | [22, 17] |
| -63.6 | -9.8 | 0 | 1 | [23, 24] |
| -63.6 | 8.2 | 1 | 0 | [4] |
| -63.6 | 9.7 | 0 | 1 | [25, 26] |
| -63.1 | -9.8 | 0 | 1 | [27] |
| -63.1 | -7.3 | 1 | 0 | [5] |
| -63.1 | -3.3 | 1 | 0 | [5] |
| -63.1 | 3.2 | 0 | 1 | [28] |
| -62.6 | -3.8 | 1 | 0 | [29] |
| -62.6 | 3.2 | 0 | 1 | [28] |
| -62.1 | -11.3 | 0 | 1 | [30] |
| -62.1 | -10.8 | 0 | 1 | [31, 30] |
| -62.1 | -9.8 | 0 | 1 | [27] |
| -62.1 | -9.3 | 0 | 1 | [18] |
| -62.1 | -6.8 | 1 | 0 | [14] |
| -62.1 | 9.2 | 1 | 1 | [4, 18] |
| -61.6 | -11.8 | 0 | 1 | [32] |
| -61.6 | -3.8 | 1 | 0 | [14] |
| -61.1 | -11.8 | 0 | 1 | [31, 33] |
| -61.1 | -3.3 | 1 | 0 | [34] |
| -61.1 | -2.8 | 1 | 0 | [20] |
| -60.6 | -13.3 | 0 | 1 | [35] |
| -60.6 | -3.3 | 1 | 1 | [36, 37] |
| -60.6 | -2.8 | 0 | 1 | [38] |
| -60.6 | 2.7 | 1 | 1 | [28, 39] |
| -60.6 | 3.7 | 1 | 0 | [40, 41] |
| -60.1 | -3.8 | 1 | 0 | [42] |
| -60.1 | -3.3 | 1 | 1 | [40, 41, 43, 44, 34, 45, 46, 47, 48, 49, 50] |
| -60.1 | -1.3 | 0 | 1 | [46, 51] |
| -59.6 | -3.3 | 1 | 1 | [52, 53, 38] |
| -59.6 | -2.8 | 0 | 1 | [54, 55] |
| -59.6 | -1.8 | 0 | 1 | [56, 38] |
| -59.6 | 3.2 | 0 | 1 | [28] |
| -59.1 | -2.8 | 0 | 1 | [57] |
| -58.6 | -3.3 | 0 | 1 | [58] |
| -56.6 | -4.3 | 1 | 0 | [5] |
| -56.6 | -2.8 | 1 | 0 | [5] |
| -56.1 | -15.8 | 0 | 1 | [59] |
| -56.1 | -9.8 | 0 | 1 | [60] |
| -56.1 | -4.3 | 1 | 0 | [61] |
| -56.1 | -2.3 | 0 | 1 | [62, 63] |
| -55.6 | -15.8 | 1 | 0 | [64] |
| -55.6 | -12.3 | 0 | 1 | [30] |
| -55.6 | -1.8 | 1 | 1 | [5, 63] |
| -54.6 | -25.3 | 1 | 1 | [65] |
| -54.6 | -2.8 | 1 | 1 | [63] |
| -54.6 | -2.3 | 1 | 1 | [29, 66, 63] |
| -54.6 | -0.8 | 0 | 1 | [63] |
| -54.1 | -24.8 | 0 | 1 | [67] |
| -54.1 | -2.3 | 1 | 0 | [5] |
| -54.1 | -1.8 | 0 | 1 | [63] |
| -53.6 | -22.8 | 1 | 0 | [68, 69] |
| -53.6 | -22.8 | 1 | 0 | [68, 69] |
| -51.1 | 0.2 | 0 | 1 | [70] |
| -49.1 | -5.3 | 0 | 1 | [71] |
| -48.6 | -17.8 | 1 | 0 | [72] |
| -48.6 | -15.8 | 0 | 1 | [73] |
| -48.6 | -12.3 | 1 | 0 | [74] |
| -48.6 | -11.8 | 1 | 0 | [75] |
| -48.6 | -10.8 | 0 | 1 | [76] |
| -48.6 | -10.3 | 1 | 0 | [75] |
| -48.6 | -1.3 | 1 | 1 | [4, 77, 61] |
| -48.1 | -21.3 | 0 | 1 | [78, 79, 80, 81, 82] |
| -48.1 | -13.3 | 1 | 0 | [83] |
| -48.1 | -7.3 | 0 | 1 | [84] |
| -47.6 | -22.3 | 0 | 1 | [85, 86] |
| -47.6 | -21.8 | 0 | 1 | [87, 88, 89, 90] |
| -47.6 | -20.3 | 0 | 1 | [91] |
| -47.6 | -1.3 | 0 | 1 | [71] |
| -47.1 | -22.8 | 0 | 1 | [92] |
| -47.1 | -21.3 | 0 | 1 | [93] |
| -47.1 | -1.8 | 0 | 1 | [71] |
| -45.1 | -21.3 | 0 | 1 | [94] |
| -44.1 | -3.3 | 0 | 1 | [95] |
| -44.1 | -2.8 | 1 | 0 | [73] |
| -43.6 | -19.8 | 0 | 1 | [96] |
| -43.6 | -4.3 | 1 | 1 | [95] |
| -43.1 | -22.8 | 0 | 1 | [97] |
| -42.6 | -21.3 | 0 | 1 | [98] |
| -42.6 | -20.8 | 0 | 1 | [99, 100] |
| -42.6 | -5.3 | 0 | 1 | [101] |
| -41.6 | -21.3 | 0 | 1 | [102] |
| -41.1 | -21.8 | 0 | 1 | [103] |
| -41.1 | -8.3 | 0 | 1 | [71] |
| -40.6 | -19.3 | 0 | 1 | [104] |
| -40.6 | -9.3 | 0 | 1 | [105, 106] |
| -40.6 | -3.8 | 0 | 1 | [65] |
| -40.1 | -3.8 | 0 | 1 | [65] |
| -39.1 | -3.8 | 1 | 1 | [107, 90, 65] |
| -38.6 | -6.3 | 0 | 1 | [65] |
| -38.6 | -4.3 | 0 | 1 | [65] |
| -38.6 | -3.8 | 0 | 1 | [108, 65] |
| -38.1 | -9.3 | 0 | 1 | [109] |
| -37.1 | -10.8 | 0 | 1 | [110, 111] |
| -36.6 | -10.3 | 1 | 1 | [93, 90, 112, 111, 113] |
| -35.1 | -8.3 | 1 | 0 | [114] |

**References**

1. Ortiz JC, Saltos N, Giacometti JC, Arrobo A, Peñafiel C, Falconi R. Alternativas alimenticias para el cultivo de *Colossoma macropomum* en jaulas flotantes. Boletín Técnico. 2007; 7: 72–81.
2. Soberon L, Mathews P, Malherios A. Hematological parameters of *Colossoma macropomum* naturally parasitized by Anacanthorus spathulatus (Monogenea: Dactylogiridae) in fish farm in the Peruvian Amazon. Int Aquat Res. 2014; 6: 251-255. doi:10.1007/s40071-014-0087-1
3. Tomalá D, Chavarría J, Ángeles B. Evaluación de la tasa de consumo de oxígeno de *Colossoma macropomum* en relación de al peso corporal y temperatura del agua. Lat Am J Aquat Res. 2014; 42: 971-979.
4. Farias IP, Torrico JP, García-Dávila C, Santos M da CF, Hrbek T, Renno JF. Are rapids a barrier for floodplain fishes of the Amazon basin? A demographic study of the keystone floodplain species *Colossoma macropomum* (Teleostei: Characiformes). Mol Phylogenet Evol. 2010; 56: 1129-1135. doi:10.1016/j.ympev.2010.03.028
5. Da Costa LRF, Barthem RB, Bittencourt MM. A pesca do tambaqui, *Colossoma macropomum*, com enfoque na área do médio Solimões, Amazonas, Brasil. Acta Amaz. 2001; 31: 449-468.
6. Anderson JT, Rojas SJ, Flecker AS. High-quality seed dispersal by fruit-eating fishes in Amazonian floodplain habitats. Oecologia. 2009; 161: 279-290. doi:10.1007/s00442-009-1371-4
7. Aldea-Guevara MI, Hargrove J, Austin JD. Diversity and geneflow in a migratory frugivorous fish: Implications for Amazonian habitat connectivity. Conserv Genet. 2013; 14: 935-942. doi:10.1007/s10592-012-0442-y
8. Hernández M, Takeuchi T, Watanabe T. Effect of Dietary Energy Sources on the Utilization of Protein by *Colossoma macropomum* Fingerlings. Fish Sci. 1995; 61: 507-511.
9. Eckmann R. Growth and Body Composition of Juvenile *Colossoma macropomum* Cuvier 1818 (Chariacoidei) Feeding on Artificial Diets. Aquaculture. 1987; 64: 293-303.
10. Delgado PM, Delgado JPM, Arenas JV, Orbe RI. Massive infestation by Perulernaea gamitanae (Crustacea: Cyclopoida: Lernaidae) in juvenile gamitana, cultured in the Peruvian Amazon. Vet Méx. 2011; 42: 59-64.
11. Santis HP, Chacón LA, Echavarria AE, Inchima SU, Molina DP, Ángel MO, et al. Caracterización de la diversidad genética en el pez *Brycon henni* (Characiformes: Characidae) en Colombia central por medio de marcadores RAPD. Rev Biol Trop. 2007; 55: 3-4.
12. De Pasquier GA, Méndez Y, Perdomo DA. Engorde experimental de cachama (*Colossoma macropomum*) en la Estación Local El Lago, estado Zulia, Venezuela. Zootec Trop. 2011; 29: 213-218.
13. Morillo MS, Visbal TB, Altuve D, Ovalles FD, Medina ALG. Valoración de dietas para alevines de *Colossoma macropomum* utilizando como fuentes proteicas harinas: de lombriz (*Eisenia foetida*), soya (*Glycine max*) y caraotas (*Phaseolus vulgaris*). Rev Chil Nutr. 2013; 40: 147-154.
14. Villacorta-Correa MA, Saint-Paul U. Structural indexes and sexual maturity of tambaqui Colossoma macropomum (Cuvier, 1818) (Characiformes: Characidae) in central Amazon, Brazil. Rev Bras Biol. 1999; 59: 637-652.
15. Poleo G, Aranbarrio JV, Mendoza L, Romero O. Cultivo de cachama blanca en altas densidades y en dos sistemas cerrados. Pesqui Agropecu Bras. 2011;46: 429–437. doi:10.1590/S0100-204X2011000400013
16. Fogel DED, Zambrano JLF, Gonzalez I. Parasitosis en *Colossoma macropomum* (Pisces: Characidae) cultivado, ocasionada por los protozoos *Ichthyophthirius multifilis* (Fouquet) y *Piscinoodinium pillulare* (Schäperclaus). Saber. 2004; 16: 3-8.
17. Salazar LEM. Biología de la reproducción y crecimiento de *Colossoma macropomum* em la Amazonía Boliviana. Universidad Mayor de San Andrés. 2004
18. Salazar Lugo R, Nathalí G, Villalobos LBB, Mairin L. Immunological response of the freshwater fish *Colossoma macropomum* as a biomarker of copper exposure. Bull Environ Contam Toxicol. 2006; 77: 925-930. doi:10.1007/s00128-006-1232-3
19. Silvano RAM, Ramires M, Zuanon J. Effects of fisheries management on fish communities in the floodplain lakes of a Brazilian Amazonian Reserve. Ecol Freshw Fish. 2009; 18: 156-166. doi:10.1111/j.1600-0633.2008.00333.x
20. Ardura A, Gomes V, Linde AR, Moreira JC, Horreo JL, Garcia-Vazquez E. The Meeting of Waters, a possible shelter of evolutionary significant units for Amazonian fish. Conserv Genet. 2013; 14: 1185-1192. doi:10.1007/s10592-013-0505-8
21. Maccord PFL, Silvano RAM, Ramires MS, Clauzet M, Begossi A. Dynamics of artisanal fisheries in two Brazilian Amazonian reserves: Implications to co-management. Hydrobiologia. 2007; 583: 365-376. doi:10.1007/s10750-006-0486-4
22. Reinert TR, Winter KA. Sustainability of harvested pacú (*Colossoma macropomum*) populations in the northeastern Bolivian Amazon. Conserv Biol. 2002;16: 1344–1351. doi:10.1046/j.1523-1739.2002.01078.x
23. Salazar-Lugo R, Pérez R, León A, Lemus M, Astudillo LR. Determinación de tiolez totales solubles en ácido en el pez *Colossoma macropomum* (Cuvier, 1818) expuesto a cadmio. Rev Científica FCV-LUZ. 2009; 19: 412–420.
24. Salazar-Lugo R, Estrella A, Oliveros A, Rojas-Villarroel E, Villalobos de B L, Lemus M. Paraquat and temperature affect nonspecific immune response of *Colossoma macropomum*. Environ Toxicol Pharmacol. 2009; 27: 321-326. doi:10.1016/j.etap.2008.11.010
25. Rojas L-M, Mata C, Oliveros A, Salazar-Lugo R. Histologia de braquias, hígado y riñón de juveniles del pez neotropical *Colossoma macropomum* (Characiformes, Characidae) expuetos a tres temperaturas. Rev da Bíologia Trop. 2013; 61: 797-806.
26. Salazar-Lugo R, Vargas A, Rojas L, Lemus M. Histopathological changes in the head kidney induced by cadmium in a neotropical fish *Colossoma macropomum*. Open Vet J. 2013; 3: 145-150.
27. Oliveira SRKS. Estudo da endofauna parasitária do tambaqui, *Colossoma macropomum*, em pisciculturas do vale do Jamari – Ro. Universidade Camilo Castelo Branco. 2014.
28. Melo Filho AA, De Oliveira HH, Dos Santos RC. Omega-6/Omega-3 and PUFA/SFA in *Colossoma macropomum* Grown in Roraima, Brazil. Orbital Electron J Chemitry. 2013; 5: 30-34.
29. Fischer C, Malta JC de O, Varella AMB. A fauna de parasitas do tambaqui, *Colossoma macropomum* (Cuvier, 1818) (Characiformes: Characidae) do médio Solimões, Estado do Amazonas (AM) e do baixo do rio Amazonas, estado do Pará (PA), e seu potencial como indicadores biológicos. Acta Amaz. 2003; 33: 651-662.
30. Jacometo CB, Barrero NML, Rodriguez‑Rodriguez MDP, Gomes PC, Povh JA, Streit Junior DP, et al. Variabilidade genética em tambaquis (Teleostei: Characidae) de diferentes regiões do Brasil. Pesqui Agropecuária Bras. 2010; 45: 481-487.
31. Lopes TS, Streit DP, Ribeiro RP, Povh JA, Lopera-Barrero NM, Vargas L, et al. Diversidade genética de estoques de reprodutores de *Colossoma macropomum*. Arq Bras Med Vet Zootec. 2009; 61: 728-735.
32. Godoi MMIDM, Engracia V, Lizama M de LAP, Takemoto RM. Parasite-host relationship between the tambaqui (*Colossoma macropomum* Cuvier 1818) and ectoparasites, collected from fish farms in the City of Rolim de Moura, State of Rondônia, Western Amazon, Brazil. Acta Amaz. 2012; 42: 515-524.
33. Varela Junior AS, Goularte KL, Alves JP, Pereira FA, Silva EF, Cardoso TF, et al. Methods of cryopreservation of Tambaqui semen, *Colossoma macropomum*. Anim Reprod Sci. 2015; 157: 71-77. doi:10.1016/j.anireprosci.2015.03.017
34. Saint-Paul U, Zuanon J, Villacorta Correa MA, García M, Fabré NN, Berger U, et al. Fish communities in central Amazonian white-and blackwater floodplains. Environ Biol Fishes. 2000; 57: 235-250.
35. Borges AF, Santos AA, De Rezende JLP, Borges M dos ACS, Ciríaco A da P, Santiago TMO. Environmental performance of aquaculture in Rondônia state, Brazil. Rev Ceres. 2015; 62: 208-214. doi:10.1590/0034-737X201562020011
36. Gonçalves C, Batista VS. Avaliação do desembarque pesqueiro efetuado em Manacapuru, Amazonas, Brasil Evaluation of the Manacapuru fishing landings, Amazonas State, Brazil. Acta Amaz. 2008; 38: 135-144.
37. Sousa RGC, Freitas CEDCF. Seasonal catch distribution of tambaqui (*Colossoma macropomum*), Characidae in a central Amazon floodplain lake: Implications for sustainable fisheries management. J Appl Ichthyol. 2011; 27: 118-121. doi:10.1111/j.1439-0426.2010.01521.x
38. Nakayama CM, Feldberg E, Bertollo LAC. Karyotype differentiation and cytotaxonomic considerations in species of Serrasalmidae (Characiformes) from the Amazon basin. Neotrop Ichthyol. 2012; 10: 53-58. doi:10.1590/S1679-62252012000100005
39. Ramos CAM. Relação das medidas de amplitude bucal de tambaqui (*Colossoma macropomum*, Cuvier, 1818) com algumas variáveis biométricas. Universidade Federal de Roraima. 2013.
40. Saint-Paul U. Investigations on the seasonal changes in the chemical composition of liver and chemical from a neotropical characoid fish *Colossoma macropomum* (Serrassalmidae). Amazoniana. 1984a; 147-158.
41. Saint-Paul U. Physiological adaptation to hypoxia of a neotropical characoid fish *Colossoma macropomum*, Serrasalmidae. Environ Biol Fishes. 1984b;11: 53-62. doi:10.1007/BF00001845
42. Sousa RGC, Freitas CEDC. The influence of flood pulse on fish communities of floodplain canals in the Middle Solimões River, Brazil. Neotrop Ichthyol. 2008;6: 249-255. doi:10.1590/S1679-62252008000200013
43. de Almeida-Val VMF, Schwantes MLB, Val AL. LDH isozymes in amazon Fish-I. Electrophoretic studies on two species from serrasalmidae family: *Mylossoma duriventris* and *Colossoma macropomum*. Comp Biochem Physiol - Part B Biochem. 1990; 95B: 77–84. doi:10.1016/0305-0491(90)90251-N
44. Santos MQDC, Oishi CA, Pereira Filho M, Lima M do AC, Ono EA, Affonso EG. Physiological response and performance of tambaqui fed with diets supplemented with Amazonian nut. Ciência Rural. 2010; 40(10): 2181-2185.
45. Arbeláez-Rojas GA, Fracalossi DM, Fim JDI. Composição Corporal de Tambaqui, *Colossoma macropomum*, e Matrinxã, *Brycon cephalus*, em Sistemas de Cultivo Intensivo, em Igarapé, e Semi-Intensivo, em Viveiros. Rev Bras Zootec. 2002; 31: 1059-1069.
46. Freitas RS, Boijink CL, Muniz AW, Dairiki JK, Inoue LAKA. Qualidade da água e perspectivas para gerenciamento ambiental dos cultivos de tambaqui no município de Rio Preto da Eva, AM. Sci Amaz. 2014; 3: 116-126.
47. Inoue LAKA, Bezerra AC, Miranda WS, Muniz AW, Boijink CDL. Cultivo de tambaqui em gaiolas de baixo volume: Efeito da densidade de estocagem na produção de biomassa. Cienc Anim Bras. 2014; 15: 437-443. doi:10.590/1089-6891v15i426758
48. Gomes LDC, Chagas EC, Martins-Junior H, Roubach R, Ono EA, Lourenço JN de P. Cage culture of tambaqui (*Colossoma macropomum*) in a central Amazon floodplain lake. Aquaculture. 2006; 253: 374-384. doi:10.1016/j.aquaculture.2005.08.020
49. Moura MAF, Farias IP, Val AL. Effects of temperature on leucocytes of *Colossoma macropomum* and *Hoplostermum littorale* (Pisces). Brazilian J Med Biol Res. 1994;27: 1589-1598.
50. Almeida NM, Franco MRB. Determination of Essential Fatty Acids in Captured and Farmed Matrinxã (*Brycon cephalus*) from the Brazilian Amazonian Area. J Am Oil Chem Soc. 2009; 83: 707-711. doi:10.1007/s11746-009-1416-1
51. Da Costa JI. Avaliação econômica e participação do plâncton no cultivo de tambaqui em viveiros com diferentes densidades de estocagem. Universidade Estadual Paulista. 2013.
52. Oliveira ACB, Soares MGM, Martinelli LA, Moreira MZ. Carbon sources of fish in an Amazonian floodplain lake. Aquat Sci. 2006; 68: 229-238. doi:10.1007/s00027-006-0808-7
53. Soares MGM, Menezes NA, Junk WJ. Adaptations of fish species to oxygen depletion in a central Amazonian floodplain lake. Hydrobiologia. 2006; 568: 353-367. doi:10.1007/s10750-006-0207-z
54. Santos L, Pereira Filho M, Sobreira C, Ituassú D, Fonseca FAL. Exigência protéica de juvenis de tambaqui (*Colossoma macropomum*) após privação alimentar. Acta Amaz. 2010; 597: 597-604.
55. Silva CR, Gomes LC, Brandão FR. Effect of feeding rate and frequency on tambaqui (*Colossoma macropomum*) growth, production and feeding costs during the first growth phase in cages. Aquaculture. 2007; 264: 135-139.doi:10.1016/j.aquaculture.2006.12.007
56. Roubach R, Gomes LC, Fonseca FAL, Val AL. Eugenol as an efficacious anaesthetic for tambaqui, *Colossoma macropomum* (Cuvier). Aquac Res. 2005; 36: 1056-1061. doi:10.1111/j.1365-2109.2005.01319.x
57. Cartonilho MM, de Jesus RS. Qualidade de cortes congelados de tambaqui cultivado. Pesqui Agropecuária Bras. 2011; 46: 344-350.doi:10.1590/S0100-204X2011000400002
58. Wood CM, Wilson RW, Gonzalez RJ, Patrick ML, Bergman H. Responses of an Amazonian Teleost, the Tambaqui (*Colossoma Macropomum*), to Low Ph in Extremely Soft Water. Wyoming Sch Repos. 1998; doi:10.1086/515977
59. Ferreira CM, Antoniassi NAB, Silva FG, Povh JA, Potença A, Moraes TCH, et al. Características histomorfométricas do intestino de juvenis de tambaqui após uso de probiótico na dieta e durante transporte. Pesqui Vet Bras. 2014; 34: 1258-1264. doi:10.1590/S0100-736X2014001200020
60. Costa G de MC, Ortis RC, De Lima MG, Casals JB, De Lima AR, Kfoury Júnior JR. Estrutura morfológica do fígado de tambaqui *Colossoma macropomum* (Cuvier, 1818). Pesqui Veterinária Bras. 2012; 32: 947-950.
61. Vieira MJAF, Carvalho MAM, Salmito-Vanderley CSB, Salgueiro CC de M, Viveiros ATM, Moura AAAN, Nunes JF. Tambaqui semen characteristics on equatorial latitude (*Colossoma macropomum*). Arch Zootec. 2011; 60(232): 1263-1270.
62. Hamoy IG, Santos S. Multiplex PCR panel of microsatellite markers for the tambaqui, *Colossoma macropomum*, developed as a tool for use in conservation and broodstock management. Genet Mol Res. 2012; 11: 141-146. doi:10.4238/2012.January.26.1
63. Aguiar J, Schneider H, Gomes F, Carneiro J, Santos S, Rodrigues LR, et al. Genetic variation in native and farmed populations of Tambaqui (*Colossoma macropomum*) in the Brazilian Amazon: Regional discrepancies in farming systems. An Acad Bras Cienc. 2013; 85: 1439-1447. doi:10.1590/0001-376520130007
64. Nupélia/ UEM/ dataset* (Available in: Veríssimo S, Pavanelli CS, Britski HA, Moreira MMM. Fish, Manso Reservior region of influence, Rio Paraguai basin, Mato Grosso State, Brazil.Check List. 2005; 1(1): 1-9).
65. Cohen SC, Kohn A. On Dactylogyridae (Monogenea) of four species of characid fishes from Brazil. Check List. 2009; 5: 351-356.
66. Martelo J, Lorenzen K, Crossa M, Mcgrath DG. Habitat associations of exploited fish species in the Lower Amazon river-floodplain system. Freshw Biol. 2008; 53: 2455-2464. doi:10.1111/j.1365-2427.2008.02065.x
67. Signor AA, Boscolo WR, Feiden A, Bittencourt F, Coldebella A, Reidel A. Fósforo na alimentação de pacus criados em tanques-rede. Arq Bras Med Vet e Zootec. 2014;39: 2336-2341. doi:10.1590/1678-5866
68. Nupélia/ UEM/ dataset* (PELD 2000-2009)
69. Nupélia/ UEM/ dataset* (PELD 2013-2014)
70. Dias-Grigório MKR. Diversidade parasitária e relação parasito-hospedeiro em *Colossoma macropomum* e seu híbrido tambatinga cultivados em Macapá, estado do Amapá. Universidade Federal do Amapá. 2013.
71. Gomes F, Schneider H, Barros C, Sampaio D, Hashimoto D, Porto-Foresti F, Sampaio I. Innovative molecular approach to the identification of *Colossoma macropomum* and its hybrids. An Acad Bras Ciênc. 2012; 84(2) 517-525.
72. Nupélia/ UEM/ dataset* (Available in: Pavanelli CS, Graça WJ, Zawadzki CH, Britski HA, Vidotti AP, Avelino GS, Veríssimo S. Fishes from the Corumbá Reservoir, Paranaíba River drainage, upper Paraná River basin, State of Goiás, Brazil. Check List. 2007; 3(1): 58-64).
73. Souza RA, Padua DMC, Oliveira RPC, Maia TCB, Souza RA, et al. Análise econômica da criação de tambaqui em tanques-rede: estudo de caso em assentamento da reforma agrária. Custos e Agronegócios. 2014; 10: 253-268. Available: [www.custoseagronegocioonline.com.br](http://www.custoseagronegocioonline.com.br)
74. Neamb/ UFT/ dataset* (Available in: Monteiro AS, Oliveira AHM, Pelicice FM. Alterações na estrutura trófica da ictiofauna: sucessos e insucessos na colonização do reservatório. In: Agostinho CS, Pelicice FM, Marques EE. Reservatório de Peixe Angical: bases ecológicas para o manejo da ictiofauna. São Carlos: RiMa Editora; 2009. p87-94).
75. Neamb/ UFT/ dataset* (Available in: Lucinda PHF, Freitas IS, Soares AB, Marques EE, Agostinho CS, Oliveira RJ. Fish, Lajeado Reservoir, rio Tocantins drainage, State of Tocantins, Brazil. Check List. 2007; 3(2): 70-83).
76. Seibert CS, Guerra-Shinohara EM, De Carvalho EG, Marques EE. Red blood cell parameters and osmotic fragility curve of *Colossoma macropomum* (Pisces, Osteichthyes, Mileinae) in captivity. Acta Sci Mar. 2001; 23: 515-520.
77. Souza AS. Análise de desenvolvimento do tambaqui, *Colossoma macropomum* (Cuvier, 1818) (Pisces, Serrasalmidae), utilizando a massa de mandioca branca Manihot esculenta (Crantz) como complemento alimentar em viveiros de pisciculturas em área de várzea. Universidade Federal do Pará. 2009.
78. Macaria M, Carneiro DJ, Larson ML, Machado CR. Influence of dietary protein intake and recombinant human somatotropin administration on growth and body composition of juvenile tambacu (a *Piaractus mesopotamicus* X *Colossoma macropomum* cross). Aquaculture. 1994; 127: 363-369.
79. Tavares-Dias M, Sandrim EFS. Características hematológicas brasileiros. I. Série vermelha e dosagens de cortisol e glicose do plasma sanguineo de espécimes de *Colossoma macropomum* em condições de cultivo. Acta Sci. 1998; 20: 157-160.
80. Tavares-Dias M, Sandrim EFS, Campos-Filho E. Características hematológicas do tambaqui *Colossoma macropomum* Cuvier (Osteichthyes, Characidae) em sistemas de monocultivo intensivo. II. Leoucócitos. Rev Bras Zool. 1999; 16(1): 175-184.
81. Tavares-Dias M, Schalch SHC, Martins ML, Onaka EM, Moraes FR. Haematological characteristics of Brazilian Teleosts. III. Parameters of the hybrid tambacu (*Piaractus mesopotamicus* Holmberg x *Colossoma macropomum* Cuvier) (Osteichthyes, Characidae). Rev Bras Zool. 2000; 17: 899-906.
82. Pádua SB, Pilarski F, Sakabe R, Dias-neto J, Chagas EC, Ishikawa MM. Heparina e K3 EDTA como anticoagulantes para tambaqui (*Colossoma macropomum* Cuvier, 1816). Acta Amaz. 2012; 42: 293-298.
83. Limnobios/ UHE São Salvador/ dataset* (KDGLuz-Agostinho, Personal Communication)
84. Chapadense PFG, Castro F de J, Almeida JA, Moron SE. Toxicity of atrazine herbicide in *Colossoma macropomum*. Rev Bras Saúde Prod An. 2009; 10: 398-405.
85. Florindo LH, Reid SG, Kalinin AL, Milsom WK, Rantin FT. Cardiorespiratory reflexes and aquatic surface respiration in the neotropical fish tambaqui (*Colossoma macropomum*): Acute responses to hypercarbia. J Comp Physiol B Biochem Syst Environ Physiol. 2004; 174: 319-328. doi:10.1007/s00360-004-0417-5
86. Corrêa CF, de Aguiar LH, Lundstedt LM, Moraes G. Responses of digestive enzymes of tambaqui (*Colossoma macropomum*) to dietary cornstarch changes and metabolic inferences. Comp Biochem Physiol - A Mol Integr Physiol. 2007; 147: 857-862. doi:10.1016/j.cbpa.2006.12.045
87. Merola N, Cantelmo OA. Growth, feed conversion and mortality of cagereared tambaqui, *Colossoma macropomum*, fed various dietary feeding regimes and protein levels. Aquaculture. 1987; 66: 223-233. doi:10.1016/0044-8486(87)90108-6
88. Merola N, Pagán-Font FA. Pond culture of the Amazon fish tambaqui, *Colossoma macropomum*: A pilot study. Aquac Eng. 1988; 7: 113-125. doi:10.1016/0144-8609(88)90009-X
89. Merola N, De Souza H. Cage Culture of the Amazon Fish Tambaqui, *Colossoma macropomum*, at Two Stocking Densities. Aquaculture. 1988; 71: 15-21.
90. Calcagnotto D, Toledo-Filho SDA. Loss of genetic variability at the transferrin locus in five hatchery stocks of tambaqui (*Colossoma macropomum*). Genet Mol Biol. 2000; 23: 127-130. doi:10.1590/S1415-47572000000100023
91. Tavares-Dias M, Martins ML, Moraes FR. Fauna parasitária de peixes oriundos de “pesque-pague” do município de Franca, São Paulo, Brasil. I. Protozoários. Rev. Bras. Zool. 2001; 18(1): 67-79.
92. Martins SN, Guzmán EC. Effect of drying method of bovine blood on the performance of growing diets for tambaqui (*Colossoma macropomum*, Cuvier 1818) in experimental culture tanks. Aquaculture. 1994; 124:335-341.
93. Calcagnotto D, De Almeida-Toledo LF, Bernardino G, Toledo-Filho SDA. Biochemical genetic characterization of F1 reciprocal hybrids between neotropical pacu (*Piaractus mesopotamicus*) and tambaqui (*Colossoma macropomum*) reared in Brazil. Aquaculture. 1999; 174: 51-57. doi:10.1016/S0044-8486(99)00005-8
94. Gonçalves ACS, Murgas LDS, Rosa PV, Navarro RD, Da Costa DV, De Alencar Teixeira E. Desempenho produtivo de tambacus alimentados com dietas suplementadas com vitamina E. Pesqui Agropecu Bras. 2010; 45: 1005-1011. doi:10.1590/S0100-204X2010000900010
95. Santos DMS, Cruz CF, Pereira DP, Alves LMC, De Moraes FR. Microbiological water quality and gill histopathology of fish from fish farming in Itapecuru-Mirim County, Maranhão State. Acta Sci Biol Sci Mar. 2012; 34: 199-205. doi:10.4025/actascibiolsci.v34i2.8460
96. Pedreira MM, Sipaúba-Tavares LH. Effect of light green and dark brown colored tanks on survival rates and development of tambaqui larvae, *Colossoma macropomum* (Osteichthyes, Serrasalmidae). Acta Sci Mar. 2001; 23: 521-525.
97. Alexandre Borges. Parâmetros de qualidade do Pacu (*Piaractus mesopotamicus*), tambaqui (*Colossoma macropomum*) e do seu híbrido eviscerado e estocado com gelo. Universidade Federal Fluminense. 2013.
98. Hancz C. Performance of the amazonian tambaqui, *Colossoma macropomum*, in pond polyculture. Aquac Eng. 1993; 12: 245-254. doi:10.1016/0144-8609(93)90015-4
99. Vasquez-Vidal et al. 2004
100. Vidal MV, Donzele JL, De Andrade DR, Dos Santos LC. Determinação da digestibilidade da matéria Seca e da proteína bruta do fubá de milho e do farelo de soja para tambaqui (*Colossoma macropomum*), utilizando-se técnicas com uso de indicadores internos e externos. Rev Bras Zootec. 2004; 33: 2193-2200. doi:10.1590/S1516-35982004000900003
101. Araripe MNBA, Araripe HGA, Lopes JB, Castro PL, Braga TEA, Ferreira AHC, Abreu MLT. Redução de proteína com suplementação de aminoácidos em rações para alevinos de tambatinga. Rev Bras Zoot. 2011; 40(9): 1845-1850.
102. Bastos PAMB. Aspectos histopatológicos de infestação por Lernaea spp (Crustáceo: Copepoda) em tambaqui (Colossoma macropomum, Cuvier, 1818). Universidade Federal Fluminense. 1995.
103. Mendonça PP. Influência do fotoperíodo no desenvolvimento de juvenis de tambaqui *Colossoma macropomum*. Universidade Estadual do Norte Fluminense. 2007.
104. Reis Neto RV. Avaliações morfométricas de juvenis de pacu (*Piaractus mesopotamicus*), tambaqui (*Colossoma macropomum*) e seus híbridos. Universidade Federal de Lavras. 2007.
105. Nunes ZMP, Lazzaro X, Peret AC. Effects of initial biomass on fish growth and fishery productivity in polyculture systems. Ciência Agrotec. 2006; 30: 1083-1090.
106. Campeche DFB, Melo JFB, Balzana L, Souza RC, Figueiredo RACR. Farelo de licuri em dietas para alevinos de tambaqui (*Colossoma Macropomum*, Cuvier, 1818). Arq Bras Med Vet e Zootec. 2014; 66: 539-545. doi:10.1590/1678-41625920
107. Molnár K, Békési L. Description of a new Myxobolus species, M. colossomatis n. sp. from the teleost *Colossoma macropomum* of the Amazon River basin. J Appl Ichthyol. 1993; 9: 57-63.
108. Saint-Paul U. Diurnal routine O2 consumption at different O2 concentrations by *Colossoma macropomum* and *Colossoma bachypomum* (Teleostei: Serrasalmidae). Camp Biochem Physiol. 1988;89A: 675-682.
109. Santos SS, Lopes JP, dos Santos Neto MA, Santos LS. Larvicultura do tambaqui em diferentes densidades de estocagem. Rev Bras Enga Pesca. 2007; 2: 18-25.
110. Maria AN, Azevedo HC, Santos JP, Silva CA, Carneiro PCF. Semen characterization and sperm structure of the Amazon tambaqui *Colossoma macropomum*. J Appl Ichthyol. 2010; 26: 779-783.
111. Maria AN, Azevedo HC, Santos JP, Carneiro PCF. Hormonal induction and semen characteristics of tambaqui *Colossoma macropomum*. Zygote. 2011; 20: 39-43. doi:10.1017/S0967199410000559
112. Almeida CAL. Caracterização e desenvolvimento embrionário e desempenho de pós-larvas de tambaqui (*Colossoma macropomum*, Cuvier, 1818) alimentados com diferentes dietas. Universidade Federal de Sergipe. 2014.
113. Maria AN, Carvalho ACM, Araújo RV, Santos JP, Carneiro PCF, Azevedo HC. Use of cryotubes for the cryopreservation of tambaqui fish semen (*Colossoma macropomum*). Cryobiology. 2015; 70: 109-114. doi:10.1016/j.cryobiol.2015.02.004
114. Viera VLA, Johnston IA. Muscle development in the tambaqui, an important Amazonian food fish. J Fish Biol. 1996; 49: 842-853.

*Partner Institutions
